# Evaluation Criteria of Noninvasive Telemonitoring for Patients With Heart Failure: Systematic Review

**DOI:** 10.2196/jmir.7873

**Published:** 2018-01-16

**Authors:** Troskah Farnia, Marie-Christine Jaulent, Olivier Steichen

**Affiliations:** ^1^ Laboratoire d’Informatique Médicale et Ingénierie des Connaissances en eSanté, Institut National de la Santé et de la Recherche Médicale Sorbonne Universités Université Paris 13, Sorbonne Paris Cité Paris France; ^2^ Department of Internal Medicine Hôpital Tenon Assistance Publique-Hôpitaux de Paris Paris France

**Keywords:** telemedicine, outcome and process assessment (health care), program evaluation, heart failure

## Abstract

**Background:**

Telemonitoring can improve heart failure (HF) management, but there is no standardized evaluation framework to comprehensively evaluate its impact.

**Objective:**

Our objectives were to list the criteria used in published evaluations of noninvasive HF telemonitoring projects, describe how they are used in the evaluation studies, and organize them into a consistent scheme.

**Methods:**

Articles published from January 1990 to August 2015 were obtained through MEDLINE, Web of Science, and EMBASE. Articles were eligible if they were original reports of a noninvasive HF telemonitoring evaluation study in the English language. Studies of implantable telemonitoring devices were excluded. Each selected article was screened to extract the description of the telemonitoring project and the evaluation process and criteria. A qualitative synthesis was performed.

**Results:**

We identified and reviewed 128 articles leading to 52 evaluation criteria classified into 6 dimensions: clinical, economic, user perspective, educational, organizational, and technical. The clinical and economic impacts were evaluated in more than 70% of studies, whereas the educational, organizational, and technical impacts were studied in fewer than 15%. User perspective was the most frequently covered dimension in the development phase of telemonitoring projects, whereas clinical and economic impacts were the focus of later phases.

**Conclusions:**

Telemonitoring evaluation frameworks should cover all 6 dimensions appropriately distributed along the telemonitoring project lifecycle. Our next goal is to build such a comprehensive evaluation framework for telemonitoring and test it on an ongoing noninvasive HF telemonitoring project.

## Introduction

Heart failure (HF) affects 26 million people worldwide, incurring direct and indirect costs of more than US $100 billion per year [1,2]. HF causes 1% to 2% of all hospitalizations, representing a major burden for patients and the health care system [3]. Number of hospitalizations is a marker of disease instability, and studies have shown that HF management can reduce this number [4].

Telemonitoring is a branch of telemedicine defined as the use of communication technologies to monitor and transmit data on the health status of patients to distant care providers [5,6]. It differs from teleconsultation, where there is a real-time interactive video or audio consultation between the patient and a distant health care provider. It also differs from tele-expertise, where a health care provider presents a patient case and gets advice from a distant colleague through a dedicated system, without direct patient involvement, in real or deferred time.

Systematic reviews of telemonitoring projects have shown reduced hospitalization rates for acute HF [7-9]. Both the European Society of Cardiology and the American Heart Association support the use of telemonitoring to improve the care of HF patients [10,11]. HF telemonitoring can involve the use of invasive or noninvasive monitoring devices. Invasive devices are implanted in the body, and data transmission is not controlled by the patient. Noninvasive devices, like weight scales or blood pressure monitors, are used, mostly by the patient, to self-monitor physiological measurements, signs, or symptoms of the disease.

The information flow is much more intricate with noninvasive than with invasive telemonitoring. First, human action is needed to handle noninvasive telemonitoring devices whereas monitored data is automatically recorded and sent by invasive telemonitoring devices. Second, patients (or family caregivers) are major actors of the noninvasive telemonitoring process whereas their involvement is minimal with invasive telemonitoring. Third, nurses and primary care physicians are usually involved in the noninvasive telemonitoring process whereas they do not take part in invasive monitoring. Fourth, as a result of these differences, noninvasive HF telemonitoring requires readiness for change, education, and training of patients and caregivers whereas invasive HF telemonitoring does not [8]. Due to these major differences, this review focuses on noninvasive HF telemonitoring.

The development of evaluation criteria for electronic tools is considered to be a critical step by the European Society of Cardiology [10]. Standard evaluation frameworks are useful to encourage systematic evaluation and get conclusive results that can be compared or aggregated across programs, allowing the analysis of determinants of success and failure for efficient resource allocation. Standard evaluation frameworks have been used for the evaluation of teleconsultation and tele-expertise [12-16]. To our knowledge, they have not yet been used for the evaluation of telemonitoring projects. Telemonitoring interventions are complex: they involve many different actors with different backgrounds (health care professional, patients and family, technicians, payers), they use technical devices, and they change the usual process of care. They can impact health care on many levels [17]: patient access to care, health and quality of life, patient and care provider education, family and care provider workload, organization of the patient care pathway, health care costs, and more. A comprehensive telemonitoring evaluation framework therefore needs to be multidimensional.

Our aim was to perform a systematic review of criteria used for the evaluation of noninvasive HF telemonitoring projects, describe how they are used in evaluation studies, and organize them into a consistent scheme.

## Methods

### Information Sources and Eligibility Criteria

We did not submit a review protocol to a prospective register. We searched Medical Literature Analysis and Retrieval System Online (MEDLINE), Excerpta Medica database (EMBASE), and Web of Science for articles published from January, 1990, to the query date (August 15, 2015) using the queries displayed in Multimedia Appendix 1. The search strategy for each bibliographic database was internally discussed, piloted, and refined by the authors but not submitted for external peer review. We checked reference lists of included articles to identify additional studies. We also took advantage of 3 Cochrane reviews on telemonitoring, published shortly after the last query date of our review, to look for missed studies [18-20].

Articles were eligible if they were original reports of a noninvasive telemonitoring project evaluation study for HF using explicit evaluation criteria. Articles were excluded if they did not contain original data (reviews, editorials, position papers, etc), were not written in English, focused on other types of telemedicine (teleconsultation, tele-expertise, etc), and if they reported invasive telemonitoring for HF through implantable devices.

### Study Selection and Data Collection

After eliminating duplicate articles, titles and abstracts were independently screened by 2 readers to exclude obviously irrelevant articles. Discordant classifications between the 2 readers were resolved through discussion. The full text of remaining articles was read by 1 investigator, who applied eligibility and exclusion criteria. The final selection was cross-checked by a second investigator. Characteristics of the telemonitoring project, characteristics of the evaluation process, and evaluation criteria were systematically abstracted by 1 investigator and cross-checked by another. The collected data are reported in Multimedia Appendix 2.

### Synthesis of Results

A preliminary list of broad evaluation dimensions was adapted by 2 investigators from previously published evaluation frameworks for telemedicine [12-16]. This categorization was then iteratively refined to meld the evaluation criteria found in each reviewed study into a consistent scheme.

## Results

### Study Selection and Characteristics

**Figure 1:**
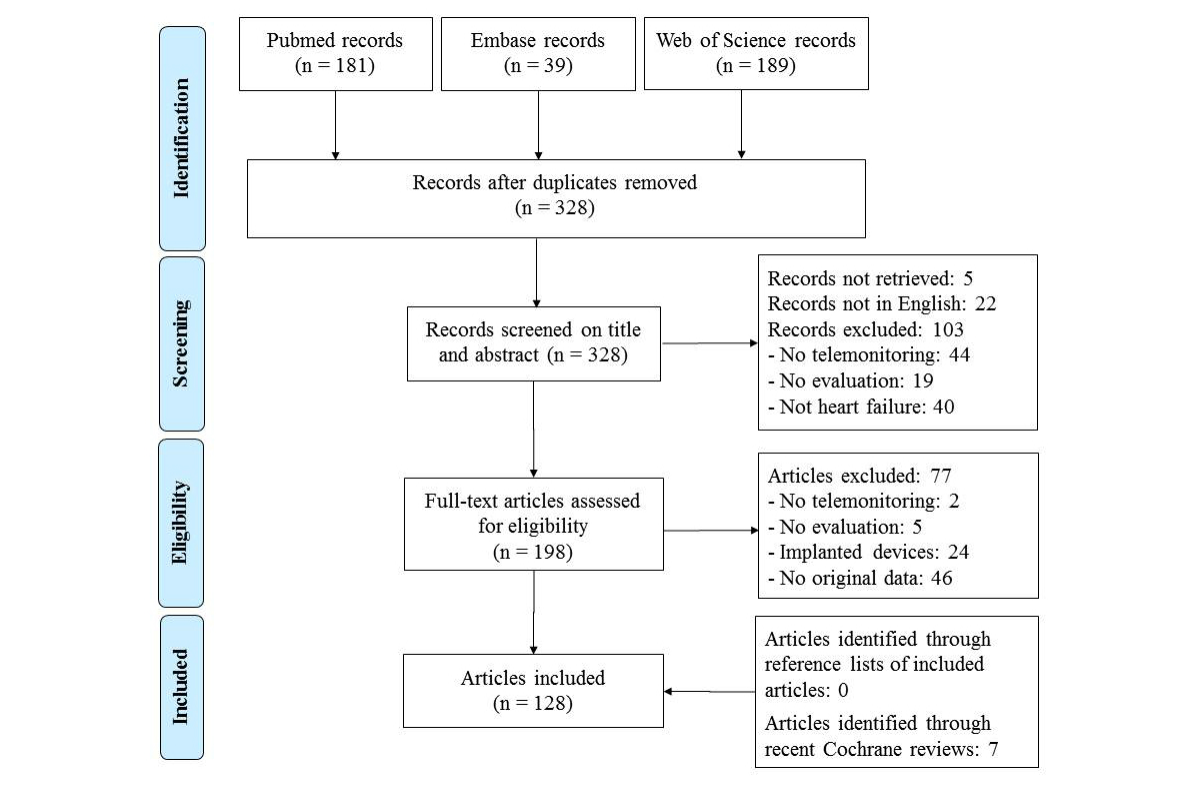
Review flowchart.

The queries of bibliographic databases identified 328 potentially eligible articles, and we included 128 articles in the review (reference list is in Multimedia Appendix 3). The review flowchart describes the process and reasons for exclusion (Figure 1). Characteristics of the 128 studies are reported in Multimedia Appendix 4, and a summary is presented in Table 1.

Europe and the United States contributed the most to the assessment of HF telemonitoring (50% of studies performed in Europe and 41% in the United States). The first study began in 1997 in the United States [21]. Europe started to carry out research to assess HF telemonitoring 3 years later [22-24].

### Telemonitoring Characteristics

Some features were highly prevalent across the telemonitoring projects: 80% were carried out by a cardiology team, and the care providers were most often HF nurses (86% of projects). Patients were actively involved in 100% of the projects, but the family of the patients, psychologists, and technicians rarely participated. In 75% of studies, patients were included in the telemonitoring program at discharge from a hospitalization for acute heart failure. The phase of the project lifecycle was clear and identifiable in 125 articles: most of these projects were in the implementation phase (59%), and no project was part of routine clinical care.

The primary monitored data in the telemonitoring projects were weight, HF symptoms, heart rate, and blood pressure. These data were transmitted via telephone (verbal communication or keypad) or Internet (mobile phone or tablet). If the monitored data fell outside predefined boundaries, a warning was triggered and led to corrective actions. However, these actions were described in only 63 articles (49% of studies).

### Evaluation Dimensions and Criteria

We retrieved 52 criteria from the 128 studies (Textbox 1) and classified them into 6 main dimensions: economic, clinical, educational, technical, user perspective, and organizational.

Most studies (95%) covered, at most, 3 dimensions, and none covered all 6 (Multimedia Appendix 5). Clinical and economic dimensions were assessed in over 70% of studies, whereas the educational, organizational, and technical dimensions were studied in less than 15% (Table 2).

The evaluation dimensions were not used homogeneously across all phases of project lifecycle. User perceptive was the most often covered dimension in the development phase with clinical and economic dimensions covered most in the later phases (implementation and integration).

Each dimension includes from 2 to 16 criteria (Textbox 1). The total number of criteria used per study ranged from 1 to 11 (Multimedia Appendix 6). The most often used evaluation criteria were cost and resource utilization (71%) and quality of life (51%) (Table 3). The criteria within the same dimension were also not used homogeneously across all phases of the project lifecycle. Concerning user perspective, for example, ease of use of the devices was more often evaluated in the development phase, whereas satisfaction with care was more often evaluated in the later phases of the project lifecycle (Table 3).

**Table 1:** Study characteristics.

Variable	Description	Value
Start of the experiment	Begins with the recruitment of the first patient.	Median: 2006 (IQR^a^ 1999, 2004) Unclear: 40% (51 studies)
Country	Country where the experiment was carried out.	Europe: 50% (64 studies) United States: 41% (53 studies) Canada: 4% (5 studies) Australia: 3% (4 studies) Asia: 1.5% (2 studies)
Care context	Location of the patients within their care pathway (after a hospitalization for an acute episode or in stable condition, followed-up by a specialist or a primary care provider).	Post-acute hospitalization: 75% (96 studies) Cardiology team: 87% (111 studies)
Telemonitoring actors	People directly involved in the telemonitoring service process (patients, health care professionals, nurses, care givers, technicians).	Patients: 100% (128 studies) Nurses: 86% (110 studies) Cardiologists: 46% (59 studies) General physicians: 26% (33 studies) Psychologists: 4% (5 studies) Families: 4% (5 studies) Technicians: 2.3% (3 studies)
Project lifecycle phase	Four phases depict the lifecycle of a project: technical development (prototype), clinical implementation (small scale experiment), integration in the clinical pathway (large scale experiment), and routine patient care.	Implementation: 59% (76 studies) Integration: 20% (26 studies) Development: 18% (23 studies) Routine care: 0% (no study) Unclear: 2.3% (3 studies)
Study design	Methodological design of the study (randomized trial, cohort study, cross-sectional study, etc).	Randomized controlled trial: 62% (79 studies) Cohort: 34% (43 studies) Cross-sectional: 3% (4 studies) Unclear: 1.5% (2 studies)
Patient age	Mean age of included patients.	Median: 69 (IQR 62.8, 74.2) years Unclear: 18% (23 studies)
Number of patients	Total number of subjects involved in the telemonitoring assessment.	Patients: median 157.5 (IQR 71, 376) Volunteers: median 17 (IQR 11, 24) Unclear: 6% (7 studies)
Number of health care professionals	Total number of health care professionals involved in the telemonitoring assessment.	Care partners: median 52 (IQR 32, 82) Nurses: median 4 (IQR 3,4) Cardiologists: median 13 (IQR 6, 20) Primary care physicians: median 260 (IQR 181, 313) Unclear: 92% (118 studies)
Associated interventions	Interventions performed to enable the home telemonitoring service (therapeutic education, training in equipment use, etc).	Therapeutic education: 32% (41 studies) Training of use of equipment: 30% (38 studies) Informed family: 4% (5 studies) Home nurse visit: 2.3% (3 studies) Technical support: 0.7% (1 study) Unclear: 46% (59 studies)
Intervention duration	Duration of the monitoring service per patient, from the first to the last data transmission.	Median: 9 (IQR 6,12) months Unclear: 10% (13 studies)

^a^IQR: interquartile range.

**Evaluation dimensions and related criteria.**

Economic

- Costs of resource utilization: days in the hospital, number of nurse visits, number of consultations with cardiologist and general practitioner, number of emergency visits, hospitalization, and readmission rate
- Cost of technical development, deployment, and maintenance of the service

Clinical

- Patient-reported outcomes: quality of life, health status, functional capacity, and activities of daily living
- Disease-oriented outcomes: mortality rate and morbidity rate
- Patient adherence to treatment: diet and medication
- Physician-adherence to guidelines

Educational

- Patient knowledge of the disease
- Patient self-care knowledge and behavior
- Primary care physician education
- Familial caregiver involvement

Technical

- Ergonomics: intuitive functions and design, quick on/off switch, and setup and configuration of the system
- Characteristics: platform connection with other devices, authentication, secure storage, maintainability, and availability of service

User perspective

- Patient perception: feelings of patient, cognitive feedback, acceptability of technology and service, reliability of information and communication technology, willingness to pay, patient motivation, social network, self-efficacy and confidence, adaptation to telephone monitoring, ease of use, access to care providers, satisfaction with new technology, compliance with new technology, and overall satisfaction with the process of care
- Care provider perception: satisfaction, utility, acceptability of technology, ease of use, compliance with new technology, and overall satisfaction with the process of care

Organizational

- Administrative: insurance policy and hospital policy
- Clinical: acceptability of heart failure nurses by general physician, heart failure nurse/ physician communication, patient/physician communication, and physician workload

**Table 2:** Coverage of evaluation dimensions across studies and phases of the project lifecycle.

Dimension	Number of studies, n (%)	Lifecycle phase	
		Development (n=23), n (%)	Implementation (n=76), n (%)	Integration (n=26), n (%)
Clinical	107 (84)	11 (48)	68 (89)	23 (88)
Economic	91 (71)	9 (39)	57 (75)	22 (85)
User perspective	55 (43)	19 (83)	31 (41)	12 (46)
Educational	18 (14)	3 (13)	10 (13)	5 (19)
Organizational	7 (5)	1 (4)	5 (7)	1 (4)
Technical	6 (4)	3 (13)	3 (4)	0 (0)

**Table 3:** Evaluation criterion most frequently used in each dimension.

Criterion	Dimension	Overall (n=128), n (%)	Lifecycle phase		
			Development (n=23), n (%)	Implementation (n=76), n (%)	Integration (n=26), n (%)
Cost of resources utilization	Economic	91 (71)	9 (39)	58 (76)	22 (85)
Quality of life	Clinical outcomes	65 (51)	8 (35)	42 (55)	14 (54)
Patient and family satisfaction with new technology	User perspective	21 (16)	7 (30)	10 (13)	3 (12)
Knowledge of disease	Educational outcomes	14 (11)	3 (13)	8 (10.5)	4 (15)
Patient and physician communication	Organizational	3 (2.3)	1 (4)	1 (1.3)	1 (4)
Reliability of transmitted data	Technical	2 (1.6)	2 (9)	0 (0)	0 (0)
Device specifications	Technical	2 (1.6)	2 (9)	0 (0)	0 (0)

## Discussion

We found 128 studies using a total of 52 evaluation criteria categorized into 6 high-level dimensions. No study covered all 6 evaluation dimensions. The evaluation dimensions were not used with the same frequency for all phases of the project lifecycle. The principle focus in the development phase was on user perspective, whereas the focus in the latter phases of the lifecycle was on the clinical and economical dimensions. The technical, organizational, and educational dimensions were poorly evaluated overall.

The 6 dimensions were derived from telemedicine assessment frameworks *Grille d'Evaluation Multidisciplinaire Santé Autonomie* (multidimensional evaluation grid for health and autonomy) [12], model for assessment of telemedicine [13], Khoja-Durrani-Scott evaluation framework [14], *Technologique, Ergonomique, Médicale, Sociale, Économique et Déontologique* (technological, ergonomic, medical, social, economic, and ethical) [15], and the 3-dimensional model [16]. These frameworks required adaptations to better fit telemonitoring. First, the role of patients and nurses is prominent in telemonitoring projects, whereas it is more limited in teleconsultation and tele-expertise. Thus, smooth collaboration must be ensured and evaluated between patients and care providers as much as between care providers themselves. Specific organizational and educational evaluation criteria are therefore needed for telemonitoring. Second, telemonitoring stands out from a technical point of view because devices are needed to gather data. The ergonomic assessment, user perception, and technical characteristics of these devices are thus key elements in the assessment of telemonitoring projects [15].

Previous evaluation frameworks are not adapted to all phases of a telemonitoring project lifecycle and do not take into account the perspective of all telemonitoring actors and external stakeholders (manufacturers, payers, etc). For example, a telemonitoring project at the first phase of its lifecycle (pilot) cannot appropriately evaluate clinical outcomes and, at the other end, proper technical evaluation is a prerequisite long before the last phase of a telemonitoring project lifecycle (routine clinical care). Criteria and indicators for a given dimension will differ across lifecycle phases. For example, the user perspective should be evaluated in all 4 phases but with different criteria: “ease of use of the system” is an appropriate criterion in the development phase, “satisfaction with new technology” in the implementation and integration phases, and “overall satisfaction with the process of care” in routine clinical care.

This review is limited by its focus on noninvasive HF telemonitoring. However, aside from disease-specific clinical outcomes, noninvasive telemonitoring services share many technical, economic, organizational, and educational features independently of the target disease. Our framework should therefore be easy to adapt to other health conditions. Our search strategy may have missed some studies that were described by other keywords. However, we found only 7 additional studies in 3 recent Cochrane reviews [18-20]. The number of missing studies is therefore likely to be low. Our 52 criteria cover all outcomes reported in previous systematic reviews on the evaluation of telemonitoring in HF [18,25] but also in other chronic diseases, such as diabetes and chronic obstructive pulmonary diseases [19,20]. We have identified broad evaluation dimensions from previously published evaluation frameworks for telemedicine projects and refined these dimensions iteratively during the review process. The 6 final dimensions accommodate all evaluation criteria used in previously published evaluation studies, but other categorization schemes are certainly possible and should be compared. The data and methods used in this review were not suited to assess the strength, limitations, relevance, and usefulness of each retrieved evaluation criterion. A follow-up to our work is needed to provide more guidance for the use of criteria in future HF telemonitoring evaluation studies.

Comprehensive telemonitoring evaluation frameworks should cover all 6 dimensions and help users choose the appropriate dimensions and evaluation criteria depending on the phase of their telemonitoring project lifecycle and the perspective of telemonitoring actors or external stakeholders they want to adopt. Our next goal is to build such a framework for noninvasive HF telemonitoring, deliberately emphasizing the technical, organizational, and educational dimensions that have been neglected by previous telemonitoring assessment studies. We will test this framework on an ongoing HF telemonitoring project.

## Appendix

Multimedia Appendix 1 Bibliographic search strategy.

Multimedia Appendix 2 Collected study characteristics.

Multimedia Appendix 3 Included papers reference list.

Multimedia Appendix 4 Detailed study characteristics.

Multimedia Appendix 5 Number of evaluation dimensions covered per study.

Multimedia Appendix 6 Number of evaluation criteria used per study.

## Abbreviations

EMBASE: Excerpta Medica database
HF: heart failure
MEDLINE: Medical Literature Analysis and Retrieval System Online
