# Supplementary material for: Evaluation Criteria of Noninvasive Telemonitoring for Patients With Heart Failure: Systematic Review
Source: J Med Internet Res. 2018 Jan 16;20(1):e16. doi: 10.2196/jmir.7873 (PMC6257336; doi:10.2196/jmir.7873)
Supplement: Multimedia Appendix 1 [file jmir_v20i1e16_app1.pdf]

Database	Access date	Search strategy
MEDLINE	14 August 2015	“Telemedicine”[Majr] AND (“Health Care Evaluation Mechanisms”[Mesh] OR “Organizational Case Studies”[Mesh] OR “Outcome and Process Assessment (Health Care)”[Mesh] OR “Patient Satisfaction”[Mesh] OR “Program Evaluation”[Mesh] OR “Technology Assessment, Biomedical”[Mesh]) AND “Heart Failure”[Majr]
EMBASE	14 August 2015	(‘Telecardiology’/exp/mj OR ‘telemonitoring’/exp/mj OR ‘telemedicine’/mj) AND (‘outcome assessment’/exp OR ‘evaluation study’/exp) AND ‘heart failure’/exp/mj AND [embase]/lim
Web of Science	14 August 2015	(TS=telemedicine OR TI=telemedicine) AND (TS=evaluation OR TI=evaluation OR TS=assessment OR TI=assessment) AND (TS=“heart failure” OR TI=”heart failure”)
