# Supplementary material for: Evaluation Criteria of Noninvasive Telemonitoring for Patients With Heart Failure: Systematic Review
Source: J Med Internet Res. 2018 Jan 16;20(1):e16. doi: 10.2196/jmir.7873 (PMC6257336; doi:10.2196/jmir.7873)
Supplement: Multimedia Appendix 2 [file jmir_v20i1e16_app2.pdf]

Topic	Description
Period of the experiment	Begins with the recruitment of the first patient and ends with the end of follow-up of the last patient.
Country	Country where the study was carried out.
Care context	Location of the patients within their care pathway (after hospitalisation for an acute episode or in stable condition, followed-up by a specialist or a primary care provider).
Centres	Multicentre or single-centre study.
Telemonitoring actors	People directly involved in the telemonitoring service process (patients, healthcare professionals, family caregivers, and technician).
External stakeholders	People indirectly involved in the telemonitoring service process (payers and managers).
Project lifecycle phase	Four phases depict the lifecycle of a project: development (prototype), implementation (small-scale experiment), integration (large-scale experiment) and sustained operation (routine patient care).
Study objective	Research question of the study.
Study design	Methodological design of the study (randomised trial, cohort study, cross sectional study, etc.).
Non-clinical inclusion criteria	All inclusion and exclusion criteria that are not directly related to the patient health status.
Patient age	Median age of included patients.
Number of patients	Total number of patients involved in the telemonitoring assessment.
Number of healthcare professionals	Total number of healthcare professionals involved in the telemonitoring assessment.
Associated intervention	Interventions performed to enable the home telemonitoring service (therapeutic education, training in equipment use, etc.).
Intervention duration	Duration of the monitoring service per patient, from the first to the last data transmission.
Monitoring devices	Connected objects used for measuring the patient data and sending them to the local transmission device.
Transmission device	Device that transmits monitoring data to the remote storage solution.
Data transmitted	Patient data sent to the remote storage solution and processed in the professional station (physiological measurements, answers to questions, etc.).
Action trigger	Data pattern indicating the need for a healthcare intervention.
Action	Healthcare intervention triggered.
Evaluation sources	Sources of data that are used for telemonitoring assessment: patients and families, healthcare professionals, medical records, administrative records, etc.
Evaluation timing	Time of assessment during the intervention period.
Evaluation dimensions	Main dimensions of evaluation: clinical, economic, technical, organisational, educational, and user perspective
Evaluation criteria	Criteria reported for telemonitoring assessment.
Perspective adopted for the evaluation	The perspective of the actors or external stakeholders for whom the evaluation is performed: patients, healthcare professionals, payers, managers, etc.
