# Supplementary material for: Evaluation Criteria of Noninvasive Telemonitoring for Patients With Heart Failure: Systematic Review
Source: J Med Internet Res. 2018 Jan 16;20(1):e16. doi: 10.2196/jmir.7873 (PMC6257336; doi:10.2196/jmir.7873)
Supplement: Multimedia Appendix 3 [file jmir_v20i1e16_app3.pdf]

1. Antonicelli R, Mazzanti I, Abbatecola AM, Parati G. Impact of home patient telemonitoring on use of beta-blockers in congestive heart failure. *Drugs Aging* 2010;**27**:801–805.

2. Antonicelli R, Testarmata P, Spazzafumo L, Gagliardi C, Bilo G, Valentini M, Olivieri F, Parati G. Impact of telemonitoring at home on the management of elderly patients with congestive heart failure. *J Telemed Telecare* 2008;**14**:300–305.

3. Baker DW, Dewalt DA, Schillinger D, Hawk V, Ruo B, Bibbins-Domingo K, Weinberger M, Macabasco-O'Connell A, Grady KL, Holmes GM, Erman B, Broucksou KA, Pignone M. The effect of progressive, reinforcing telephone education and counseling versus brief educational intervention on knowledge, self-care behaviors and heart failure symptoms. *J Card Fail* 2011;**17**:789–796.

4. Baker LC, Macaulay DS, Sorg RA, Diener MD, Johnson SJ, Birnbaum HG. Effects of care management and telehealth: a longitudinal analysis using medicare data. *J Am Geriatr Soc* 2013;**61**:1560–1567.

5. Balk AH, Davidse W, Dommelen P van, Klaassen E, Caliskan K, Burgh P van der, Leenders CM. Tele-guidance of chronic heart failure patients enhances knowledge about the disease. A multi-centre, randomised controlled study. *Eur J Heart Fail* 2008;**10**:1136–1142.

6. Bekelman DB, Plomondon ME, Carey EP, Sullivan MD, Nelson KM, Hattler B, McBryde CF, Lehmann KG, Gianola K, Heidenreich PA, Rumsfeld JS. Primary Results of the Patient-Centered Disease Management (PCDM) for Heart Failure Study: A Randomized Clinical Trial. *JAMA Intern Med* 2015;**175**:725–732.

7. Benatar D, Bondmass M, Ghitelman J, Avitall B. Outcomes of chronic heart failure. *Arch Intern Med* 2003;**163**:347–352.

8. Bernocchi P, Scalvini S, Tridico C, Borghi G, Zanaboni P, Masella C, Glisenti F, Marzegalli M. Healthcare Continuity From Hospital to Territory in Lombardy: TELEMACO Project. *American Journal of Managed Care* 2012;**18**:E101–E108.

9. Bikdeli B., Wayda B., Bao H., Ross J.S., Xu X., Chaudhry S.I., Spertus J.A., Bernheim S.M., Lindenauer P.K., Krumholz H.M. Place of residence and outcomes of patients with heart failure: Analysis from the telemonitoring to improve heart failure outcomes trial. *Circ Cardiovasc Qual Outcomes* 2014;**7**:749–756.

10. Bisio I, Lavagetto F, Marchese M, Sciarrone A. A smartphone-centric platform for remote health monitoring of heart failure. *International Journal of Communication Systems* 2015;**28**:1753–1771.

11. Black JT, Romano PS, Sadeghi B, Auerbach AD, Ganiats TG, Greenfield S, Kaplan SH, Ong MK. A remote monitoring and telephone nurse coaching intervention to reduce

readmissions among patients with heart failure: study protocol for the Better Effectiveness After Transition - Heart Failure (BEAT-HF) randomized controlled trial. *Trials* 2014;**15**.

12. Blum K, Gottlieb SS. The effect of a randomized trial of home telemonitoring on medical costs, 30-day readmissions, mortality, and health-related quality of life in a cohort of community-dwelling heart failure patients. *J Card Fail* 2014;**20**:513–521.

13. Bohme S, Geiser C, Muhlenhoff T, Holtmann J, Renneberg B. Telephone counseling for patients with chronic heart failure: results of an evaluation study. *Int J Behav Med* 2012;**19**:288–297.

14. Bowles KH, Holland DE, Horowitz DA. A comparison of in-person home care, home care with telephone contact and home care with telemonitoring for disease management. *J Telemed Telecare* 2009;**15**:344–350.

15. Boyne JJJ, Van Asselt ADI, Gorgels APM, Steuten LMG, De Weerd G, Kragten J, Vrijhoef HJM. Cost-effectiveness analysis of telemonitoring versus usual care in patients with heart failure: the TEHAF-study. *Journal of Telemedicine and Telecare* 2013;**19**:242–248.

16. Boyne JJJ, Vrijhoef HJM, Crijns HJGM, De Weerd G, Kragten J, Gorgels APM. Tailored telemonitoring in patients with heart failure: results of a multicentre randomized controlled trial. *Eur J Heart Fail* 2012;**14**.

17. Boyne JJJ, Vrijhoef HJM, Wit R de, Gorgels APM. Telemonitoring in patients with heart failure, the TEHAF study: Study protocol of an ongoing prospective randomised trial. *International Journal of Nursing Studies* 2011;**48**:94–99.

18. Boyne JJJ, Vrijhoef HJM, Spreeuwenberg M, De Weerd G, Kragten J, Gorgels APM. Effects of tailored telemonitoring on heart failure patients' knowledge, self-care, self-efficacy and adherence: a randomized controlled trial. *Eur J Cardiovasc Nurs* 2014;**13**:243–252.

19. Bradford WD, Kleit A, Krousel-Wood MA, Re RM. Comparing willingness to pay for telemedicine across a chronic heart failure and hypertension population. *Telemed J E Health* 2005;**11**:430–438.

20. Browning SV, Clark RC, Poff RM, Todd D. Telehealth monitoring: a smart investment for home care patients with heart failure? *Home healthcare nurse* 2011;**29**:368–374.

21. Capomolla S, et al. Heart failure case disease management program: a pilot study of home telemonitoring versus usual care. *European Heart Journal* 2004;**6**:91–98.

22. Cartwright M, Hirani SP, Rixon L, Beynon M, Doll H, Bower P, Bardsley M, Steventon A, Knapp M, Henderson C, Rogers A, Sanders C, Fitzpatrick R, Barlow J, Newman SP.

Effect of telehealth on quality of life and psychological outcomes over 12 months (Whole Systems Demonstrator telehealth questionnaire study): nested study of patient reported outcomes in a pragmatic, cluster randomised controlled trial. *Bmj-british Medical Journal* 2013;**346**:f653.

23. Chaudhry SI, Barton B, Mattera J, Spertus J, Krumholz HM. Randomized trial of Telemonitoring to Improve Heart Failure Outcomes (Tele-HF): study design. *J Card Fail* 2007;**13**:709–714.

24. Chaudhry SI, Mattera JA, Curtis JP, Spertus JA, Herrin J, Lin Z, Phillips CO, Hodshon BV, Cooper LS, Krumholz HM. Telemonitoring in Patients with Heart Failure. *New England Journal of Medicine* 2010;**363**:2301–2309.

25. Chen Y-. H, Ho Y-. L, Huang H-. C, Wu H-. W, Lee C-. Y, Hsu T-. P, Cheng C-. L, Chen M-. F. Assessment of the Clinical Outcomes and Cost-effectiveness of the Management of Systolic Heart Failure in Chinese Patients Using a Home-based Intervention. *Journal of International Medical Research* 2010;**38**:242–252.

26. Clark R.A., Yallop J.J., Piterman L., Croucher J., Tonkin A., Stewart S., Krum H. Adherence, adaptation and acceptance of elderly chronic heart failure patients to receiving healthcare via telephone-monitoring. *Eur J Heart Fail* 2007;**9**:1104–1111.

27. Cleland JGF, Louis AA, Rigby AS, Janssens U, Balk AHMM. Noninvasive home telemonitoring for patients with heart failure at high risk of recurrent admission and death: the Trans-European Network-Home-Care Management System (TEN-HMS) study. *J Am Coll Cardiol* 2005;**45**:1654–1664.

28. Cole SA, Farber NC, Weiner JS, Sulfaro M, Katzelnick DJ, Blader JC. Double-disease management or one care manager for two chronic conditions: pilot feasibility study of nurse telephonic disease management for depression and congestive heart failure. *Disease management : DM* 2006;**9**:266–276.

29. Copeland LA, Berg GD, Johnson DM, Bauer RL. An intervention for VA patients with congestive heart failure. *Am J Manag Care* 2010;**16**:158–165.

30. Cui Y, Doupe M, Katz A, Nyhof P, Forget EL. Economic evaluation of Manitoba Health Lines in the management of congestive heart failure. *Healthcare policy = Politiques de sante* 2013;**9**:36–50.

31. Dansky KH, Vasey J, Bowles K. Impact of telehealth on clinical outcomes in patients with heart failure. *Clinical nursing research* 2008;**17**:182–199.

32. Dar O., Riley J., Chapman C., Dubrey S.W., Morris S., Rosen S.D., Roughton M., Cowie M.R. A randomized trial of home telemonitoring in a typical elderly heart failure population in North West London: Results of the Home-HF study. *Eur J Heart Fail* 2009;**11**:319–325.

33. Dendale P, De Keulenaer G, Troisfontaines P, Weytjens C, Mullens W, Elegeert I, Ector B, Houbrechts M, Willekens K, Hansen D. Effect of a telemonitoring-facilitated collaboration between general practitioner and heart failure clinic on mortality and rehospitalization rates in severe heart failure: the TEMA-HF 1 (TElemonitoring in the MAnagement of Heart Failure) study. *Eur J Heart Fail* 2012;**14**:333–340.

34. Dierckx R, Houben R, Goethals M, Verstreken S, Bartunek J, Saeys R, De Proft M, Boel E, Vanderheyden M. Integration of remote monitoring of device diagnostic parameters into a multidisciplinary heart failure management program. *International Journal of Cardiology* 2014;**172**:606–607.

35. Domingo M, Lupon J, Gonzalez B, Crespo E, Lopez R, Ramos A, Urrutia A, Pera G, Verdu JM, Bayes-Genis A. Evaluation of a telemedicine system for heart failure patients: feasibility, acceptance rate, satisfaction and changes in patient behavior: results from the CARME (CAtalan Remote Management Evaluation) study. *European Journal of Cardiovascular Nursing* 2012;**11**:410–418.

36. Domingo M, Lupon J, Gonzalez B, Crespo E, Lopez R, Ramos A, Urrutia A, Pera G, Verdu JM, Bayes-Genis A. Noninvasive Remote Telemonitoring for Ambulatory Patients With Heart Failure: Effect on Number of Hospitalizations, Days in Hospital, and Quality of Life. CARME (CAtalan Remote Management Evaluation) Study. *Revista Espanola De Cardiologia* 2011;**64**:277–285.

37. Dunagan WC, Littenberg B, Ewald GA, Jones CA, Emery VB, Waterman BM, Silverman DC, Rogers JG. Randomized trial of a nurse-administered, telephone-based disease management program for patients with heart failure. *Journal of Cardiac Failure* 2005;**11**:358–365.

38. Dwinger S, Dirmaier J, Herbarth L, Koenig H-H, Eckardt M, Kriston L, Bermejo I, Haerter M. Telephone-based health coaching for chronically ill patients: study protocol for a randomized controlled trial. *Trials* 2013;**14**:337.

39. Ferrante D, Varini S, Macchia A, Soifer S, Badra R, Nul D, Grancelli H, Doval H. Long-term results after a telephone intervention in chronic heart failure: DIAL (Randomized Trial of Phone Intervention in Chronic Heart Failure) follow-up. *J Am Coll Cardiol* 2010;**56**:372–378.

40. Finkelstein J, Wood J, Cha E, Orlov A, Dennison C. Feasibility of congestive heart failure telemanagement using a wii-based telecare platform. *Conf Proc IEEE Eng Med Biol Soc* 2010;**2010**:2211–2214.

41. Finkelstein SM, Speedie SM, Potthoff S. Home telehealth improves clinical outcomes at lower cost for home healthcare. *Telemedicine Journal and E-health* 2006;**12**:128–136.

42. Gambetta M, Dunn P, Nelson D, Herron B, Arena R. Impact of the implementation of telemanagement on a disease management program in an elderly heart failure cohort.

Progress in cardiovascular nursing 2007;**22**:196–200.

43. Gellis ZD, Kenaley B, McGinty J, Bardelli E, Davitt J, Ten Have T. Outcomes of a telehealth intervention for homebound older adults with heart or chronic respiratory failure: a randomized controlled trial. *The Gerontologist* 2012;**52**:541–552.

44. Giordano A, Scalvini S, Zanelli E, Corra U, Longobardi GL, Ricci VA, Baiardi P, Glisenti F. Multicenter randomised trial on home-based telemanagement to prevent hospital readmission of patients with chronic heart failure. *Int J Cardiol* 2009;**131**:192–199.

45. Giordano A, Scalvini S, Paganoni AM, Baraldo S, Frigerio M, Vittori C, Borghi G, Marzegalli M, Agostoni O. Home-Based Telesurveillance Program in Chronic Heart Failure: Effects on Clinical Status and Implications for 1-Year Prognosis. *Telemedicine and E-health* 2013;**19**:605–612.

46. Giordano A, Zanelli E, Scalvini S. Home-based telemanagement in chronic heart failure: an 8-year single-site experience. *J Telemed Telecare* 2011;**17**:382–386.

47. Goldberg L, et al. Randomized trial of a daily electonic home monitoring system in patients with advanced heart failure: the weight monitoring in heart failure (WHARF) trial. *American Heart Journal* 2003;**146**:705–712.

48. Gonzalez B, Domingo M, Lupon J, Lopez R, Ramos A, Crespo E, Urrutia A, Cano L, Diez C, Valenzuela J. Changes in patients' behaviour and impact on quality of life with the use of telemedicine (Motiva-Philips) in an heart failure unit: the CARME study (CAtalan Remote Management Evaluation). *European Heart Journal* 2010;**31**:233–233.

49. Gund A, Ekman I, Lindecrantz K, Sjoqvist BA, Staaf EL, Thorneskold N. Design evaluation of a home-based telecare system for Chronic Heart Failure patients. *Conference proceedings : . Annual International Conference of the IEEE Engineering in Medicine and Biology Society IEEE Engineering in Medicine and Biology Society Annual Conference* 2008;**2008**:5851–5854.

50. Guzman-Clark JRS, Servellen G van, Chang B, Mentes J, Hahn TJ. Predictors and outcomes of early adherence to the use of a home telehealth device by older veterans with heart failure. *Telemed J E Health* 2013;**19**:217–223.

51. Jehn M, Prescher S, Koehler K, Haehling S von, Winkler S, Deckwart O, Honold M, Sechtem U, Baumann G, Halle M, Anker SD, Koehler F. Tele-accelerometry as a novel technique for assessing functional status in patients with heart failure: Feasibility, reliability and patient safety. *International Journal of Cardiology* 2013;**168**:4723–4728.

52. Jerant AF, Azari R, Nesbitt TS. Reducing the cost of frequent hospital admissions for congestive heart failure: a randomized trial of a home telecare intervention. *Med Care* 2001;**39**:1234–1245.

53. Jerant AF, Azari R, Martinez C, Nesbitt TS. A randomized trial of telenursing to reduce hospitalization for heart failure: patient-centered outcomes and nursing indicators. *Home health care services quarterly* 2003;**22**:1–20.

54. Johnston G, Weatherburn G. Automated weight monitoring in chronic heart failure: the excluded majority. *J Telemed Telecare* 2010;**16**:190–192.

55. Kashem A, Droogan MT, Santamore WP, Wald JW, Bove AA. Managing heart failure care using an internet-based telemedicine system. *J Card Fail* 2008;**14**:121–126.

56. Kashem A., Droogan M.T., Santamore W.P., Wald J.W., Marble J.F., Cross R.C., Bove A.A. Web-based internet telemedicine management of patients with heart failure. *Telemedicine J e-Health* 2006;**12**:439–447.

57. Kastner P, Morak J, Modre R, Kollmann A, Ebner C, Fruhwald FM, Schreier G. Innovative telemonitoring system for cardiology: from science to routine operation. *Applied Clinical Informatics* 2010;**1**:165–176.

58. Kessing D, Denollet J, Widdershoven J, Kupper N. Investigating a TELEmedicine solution to improve MEDication adherence in chronic Heart Failure (TELEMED-HF): study protocol for a randomized controlled trial. *Trials* 2011;**12**.

59. Kobb R, Hoffman N, Lodge R, Kline S. Enhancing elder chronic care through technology and care coordination: Report from a pilot. *Telemedicine Journal and E-health* 2003;**9**:189–195.

60. Koehler F, Winkler S, Schieber M, Sechtem U, Stangl K, Bohm M, Boll H, Baumann G, Honold M, Koehler K, Gelbrich G, Kirwan B-A, Anker SD. Impact of remote telemedical management on mortality and hospitalizations in ambulatory patients with chronic heart failure: the telemedical interventional monitoring in heart failure study. *Circulation* 2011;**123**:1873–1880.

61. Koehler F, Winkler S, Schieber M, Sechtem U, Stangl K, Bohm M, Boll H, Kim SS, Koehler K, Lucke S, Honold M, Heinze P, Schweizer T, Braecklein M, Kirwan B-A, Gelbrich G, Anker SD. Telemedical Interventional Monitoring in Heart Failure (TIM-HF), a randomized, controlled intervention trial investigating the impact of telemedicine on mortality in ambulatory patients with heart failure: study design. *Eur J Heart Fail* 2010;**12**:1354–1362.

62. Koehler F, Winkler S, Schieber M, Sechtem U, Stangl K, Bohm M, Brouwer S de, Perrin E, Baumann G, Gelbrich G, Boll H, Honold M, Koehler K, Kirwan B-A, Anker SD. Telemedicine in heart failure: pre-specified and exploratory subgroup analyses from the TIM-HF trial. *Int J Cardiol* 2012;**161**:143–150.

63. Konstam V, Gregory D, Chen J, Weintraub A, Patel A, Levine D, Venesy D, Perry K, Delano C, Konstam MA. Health-Related Quality of Life in a Multicenter Randomized

Controlled Comparison of Telephonic Disease Management and Automated Home Monitoring in Patients Recently Hospitalized With Heart Failure: SPAN-CHF II Trial. *Journal of Cardiac Failure* 2011;**17**:151–157.

64. Krum H, Forbes A, Yallop J, Driscoll A, Croucher J, Chan B, Clark R, Davidson P, Huynh L, Kasper EK, Hunt D, Egan H, Stewart S, Piterman L, Tonkin A. Telephone Support to Rural and Remote Patients with Heart Failure: The Chronic Heart Failure Assessment by Telephone (CHAT) study. *Cardiovascular Therapeutics* 2013;**31**:230–237.

65. Kulshreshtha A., Kvedar J.C., Goyal A., Halpern E.F., Watson A.J. Use of remote monitoring to improve outcomes in patients with heart failure: A pilot trial. *Int J Telemed Appl* 2010;

66. Kurtz B, et al. Automated home telephone self-monitoring reduces hospitalization in patients with advanced heart failure. *Journal of telemedicine and telecare* 2011;**17**:298–302.

67. Ledwidge MT, O'Hanlon R, Lalor L, Travers B, Edwards N, Kelly D, Voon V, McDonald KM. Can individualized weight monitoring using the HeartPhone algorithm improve sensitivity for clinical deterioration of heart failure? *European Journal of Heart Failure* 2013;**15**:447–455.

68. Lind L, Karlsson D. Digital pen-based telemonitoring of elderly heart failure patients. *Stud Health Technol Inform* 2013;**192**.

69. Lind L, Karlsson D. Telehealth for 'the digital illiterate'–elderly heart failure patients experiences. *Studies in health technology and informatics* 2014;**205**:353–357.

70. Lobodzinski SS, Jadalla AA. Integrated heart failure telemonitoring system for homecare. *Cardiol J* 2010;**17**:200–204.

71. Lusignan S, Wells S, Johnson P, Meredith K, Leatham E. Compliance and effectiveness of 1 year's home telemonitoring. The report of a pilot study of patients with chronic heart failure. *European Journal of Heart Failure* 2001;**3**:723–730.

72. Lynga P, Persson H, Hagg-Martinell A, Hagglund E, Hagerman I, Langius-Eklof A, Rosenqvist M. Weight monitoring in patients with severe heart failure (WISH). A randomized controlled trial. *Eur J Heart Fail* 2012;**14**:438–444.

73. MacKenzie E, Smith A, Angus N, Menzies S, Brulisauer F, Leslie SJ. Mixed-method exploratory study of general practitioner and nurse perceptions of a new community based nurse-led heart failure service. *Rural and Remote Health* 2010;**10**:1510.

74. Madigan E, et al. Home health care with telemonitoring improves health status for older adults with heart failure. *Home health care serv Q* 2013;**32**:57–74.

75. Martin-Lesende I, Orruno E, Cairo C, Bilbao A, Asua J, Romo MI, Vergara I, Bayon JC, Abad R, Reviriego E, Larranaga J. Assessment of a primary care-based telemonitoring intervention for home care patients with heart failure and chronic lung disease. The TELBIL study. *Bmc Health Services Research* 2011;**11**:56.

76. Masella C, Zanaboni P, Borghi G, Castelli A, Marzegalli M, Tridico C. Introduction of a telemonitoring service for patients affected by Chronic Heart Failure. *2009 11th International Conference On E-health Networking, Applications and Services (healthcom 2009)* 2009;IEEE Communications Soc; IEEE NSW Australia.

77. Morak J, Kumpusch H, Hayn D, Leitner M, Scherr D, Fruhwald FM, Schreier G. Near Field Communication-based telemonitoring with integrated ECG recordings. *Applied Clinical Informatics* 2011;**2**:481–498.

78. Morguet AJ, Kuhnelt P, Kallel A, Jaster M, Schultheiss H-P. Impact of telemedical care and monitoring on morbidity in mild to moderate chronic heart failure. *Cardiology* 2008a;**111**:134–139.

79. Morguet AJ, Kuhnelt P, Kallel A, Rauch U, Schultheiss H-P. Utilization of telemedicine by heart disease patients following hospitalization. *J Telemed Telecare* 2008b;**14**:178–181.

80. Mortara A., Pinna G.D., Johnson P., Maestri R., Capomolla S., La Rovere M.T., Ponikowski P., Tavazzi L., Sleight P. Home telemonitoring in heart failure patients: The HHH study (Home or Hospital in Heart Failure). *Eur J Heart Fail* 2009;**11**:312–318.

81. Moyer-Knox D, Mueller TM, Vuckovic K, Mischke L, Williams RE. Remote titration of carvedilol for heart failure patients by advanced practice nurses. *Journal of Cardiac Failure* 2004;**10**:219–224.

82. Mueller TM, Vuckovic KM, Knox DA, Williams RE. Telemanagement of heart failure: A diuretic treatment algorithm for advanced practice nurses. *Heart & Lung* 2002;**31**:340–347.

83. Pedone C., Rossi F.F., Cecere A., Costanzo L., Antonelli Incalzi R. Efficacy of a physician-led multiparametric telemonitoring system in very old adults with heart failure. *J Am Geriatr Soc* 2015;**63**:1175–1180.

84. Pekmezaris R, Mitzner I, Pecinka KR, Nouryan CN, Lesser ML, Siegel M, Swiderski JW, Moise G, Younker RS, Smolich K. The impact of remote patient monitoring (telehealth) upon Medicare beneficiaries with heart failure. *Telemed J E Health* 2012;**18**:101–108.

85. Piette JD, Gregor MA, Share D, Heisler M, Bernstein SJ, Koelling T, Chan P. Improving heart failure self-management support by actively engaging out-of-home caregivers: results of a feasibility study. *Congest Heart Fail* 2008;**14**:12–18.

86. Piette JD, Rosland A-M, Marinec NS, Striplin D, Bernstein SJ, Silveira MJ. Engagement With Automated Patient Monitoring and Self-Management Support Calls Experience With a Thousand Chronically Ill Patients. *Medical Care* 2013;**51**:216–223.

87. Piotrowicz E., Baranowski R., Bilinska M., Stepnowska M., Piotrowska M., Wójcik A., Korewicki J., Chojnowska L., Malek L.A., Klopotowski M., Piotrowski W., Piotrowicz R. A new model of home-based telemonitored cardiac rehabilitation in patients with heart failure: Effectiveness, quality of life, and adherence. *Eur J Heart Fail* 2010;**12**:164–171.

88. Prescher S, Deckwart O, Winkler S, Koehler K, Honold M, Koehler F. Telemedical care: feasibility and perception of the patients and physicians: a survey-based acceptance analysis of the Telemedical Interventional Monitoring in Heart Failure (TIM-HF) trial. *Eur J Prev Cardiol* 2013;**20**.

89. Quinn C. Low-technology heart failure care in home health: improving patient outcomes. *Home Healthc Nurse* 2006;**24**:533–540.

90. Radhakrishna K, Bowles K, Zettek-Sumner A. Contributors to frequent telehealth alerts including false alerts for patients with heart failure: a mixed methods exploration. *Appl Clin Inform* 2013;**4**:465–475.

91. Ramaekers B, Janssen-Boyne J, Gorgels A, Vrijhoef H. Adherence among telemonitored patients with heart failure to pharmacological and nonpharmacological recommendations. *Telemed J E Health* 2009;**15**:517–524.

92. Riegel B, Carlson B, Kopp Z, LePetri B, Glaser D, Unger A. Effect of a standardized nurse case-management telephone intervention on resource use in patients with chronic heart failure. *Archives of Internal Medicine* 2002;**162**:705–712.

93. Riegel B, Carlson B, Glaser D, Romero T. Randomized controlled trial of telephone case management in Hispanics of Mexican origin with heart failure. *J Card Fail* 2006;**12**:211–219.

94. Riley WT, Keberlein P, Sorenson G, Mohler S, Tye B, Ramirez AS, Carroll M. Program Evaluation of Remote Heart Failure Monitoring: Healthcare Utilization Analysis in a Rural Regional Medical Center. *Telemedicine and E-health* 2015;**21**:157–162.

95. Rosenman MB, Holmes AM, Ackermann RT, Murray MD, Doebbeling CC, Katz B, Li JJ, Zillich A, Prescott VM, Downs SM, Inui TS. The Indiana Chronic Disease Management Program. *Milbank Quarterly* 2006;**84**:135–163.

96. Roth A, Kajiloti I, Elkayam I, Sander J, Kehati M, Golovner M. Telecardiology for patients with chronic heart failure: the 'SHL' experience in Israel. *International Journal of Cardiology* 2004;**97**:49–55.

97. Saxon LA, Boehmer JP, Neuman S, Mullin CM. Remote Active Monitoring in Patients with Heart Failure (RAPID-RF): design and rationale. *J Card Fail* 2007;**13**:241–246.

98. Scalvini S, Zanelli E, Paletta L, Benigno M, Domeneghini D, De Giuli F, Giordano A, Glisenti F. Chronic heart failure home-based management with a telecardiology system: a comparison between patients followed by general practitioners and by a cardiology department. *J Telemed Telecare* 2006;**12 Suppl 1**:46–48.

99. Scalvini S, Zanelli E, Volterrani M, Martinelli G, Baratti D, Buscaya O, Baiardi P, Glisenti F, Giordano A. A pilot study of nurse-led, home-based telecardiology for patients with chronic heart failure. *J Telemed Telecare* 2004;**10**:113–117.

100. Scherr D, Zweiker R, Kollmann A, Kastner P, Schreier G, Fruhwald FM. Mobile phone-based surveillance of cardiac patients at home. *J Telemed Telecare* 2006;**12**:255–261.

101. Scherr D, Kastner P, Kollmann A, Hallas A, Auer J, Krappinger H, Schuchlenz H, Stark G, Grander W, Jakl G, Schreier G, Fruhwald FM. Effect of home-based telemonitoring using mobile phone technology on the outcome of heart failure patients after an episode of acute decompensation: randomized controlled trial. *J Med Internet Res* 2009;**11**.

102. Schneider NM. Managing congestive heart failure using home telehealth. *Home Healthc Nurse* 2004;**22**:719–722.

103. Schofield RS, Kline SE, Schmalfuss CM, Carver HM, Aranda JM, Pauly DF, Hill JA, Neugaard BI, Chumbler NR. Early outcomes of a care coordination-enhanced Telehome Care Program for elderly veterans with chronic heart failure. *Telemedicine Journal and E-health* 2005;**11**:20–27.

104. Schwarz KA, Mion LC, Hudock D, Litman G. Telemonitoring of heart failure patients and their caregivers: a pilot randomized controlled trial. *Prog Cardiovasc Nurs* 2008;**23**.

105. Seto E, Leonard KJ, Cafazzo JA, Barnsley J, Masino C, Ross HJ. Mobile phone-based telemonitoring for heart failure management: a randomized controlled trial. *J Med Internet Res* 2012;**14**.

106. Seto E, Leonard KJ, Masino C, Cafazzo JA, Barnsley J, Ross HJ. Attitudes of heart failure patients and health care providers towards mobile phone-based remote monitoring. *J Med Internet Res* 2010;**12**.

107. Smith B, Hughes-Cromwick PF, Forkner E, Galbreath AD. Cost-effectiveness of telephonic disease management in heart failure. *Am J Manag Care* 2008;**14**:106–115.

108. Sohn S, Helms TM, Pelleter JT, Muller A, Krottinger AI, Schoffski O. Costs and benefits of personalized healthcare for patients with chronic heart failure in the care and education program 'Telemedicine for the Heart'. *Telemed J E Health* 2012;**18**.

109. Soran O, et al. A randomized clinical trial of the clinical effects of enhanced heart failure monitoring using a computer based telephonic monitoring system in older monitorities and women. *Journal of cardiac failure* 2008;**14**:711–717.

110. Spaeder J, Najjar SS, Gerstenblith G, Hefter G, Kern L, Palmer JG, Gottlieb SH, Kasper EK. Rapid titration of carvedilol in patients with congestive heart failure: a randomized trial of automated telemedicine versus frequent outpatient clinic visits. *Am Heart J* 2006;**151**.

111. Spinsante S, Antonicelli R, Mazzanti I, Gambi E. Technological approaches to remote monitoring of elderly people in cardiology: a usability perspective. *International journal of telemedicine and applications* 2012;**2012**:104561–104561.

112. Stroetmann KA, Stroetmann VN, Westerteicher C. Implementation of TeleCare services: Benefit assessment and organisational models. *Integration of Health Telematics Into Medical Practice* 2003;**97**:131–141.

113. Suchy C, Massen L, Rognmo O, Van Craenenbroeck EM, Beckers P, Kraigher-Krainer E, Linke A, Adams V, Wisloff U, Pieske B, Halle M. Optimising exercise training in prevention and treatment of diastolic heart failure (OptimEx-CLIN): rationale and design of a prospective, randomised, controlled trial. *Eur J Prev Cardiol* 2014;**21**.

114. Svagard I, Austad HO, Seeberg T, Vedum J, Liverud A, Mathiesen BM, Keller B, Bendixen OC, Osborne P, Strisland F. A usability study of a mobile monitoring system for congestive heart failure patients. *Studies in health technology and informatics* 2014;**205**:528–532.

115. Venter A, Burns R, Hefford M, Ehrenberg N. Results of a telehealth-enabled chronic care management service to support people with long-term conditions at home. *Journal of Telemedicine and Telecare* 2012;**18**:172–175.

116. Villani A, et al. Clinical and psychological telemonitoring and telecare of high risk heart failure patients. *Journal of telemedicine and telecare* 2014;**20**:468–475.

117. Vries AE, Wal MH van der, Bedijn W, Jong RM de, Dijk RB van, Hillege HL, Jaarsma T. Follow-up and treatment of an instable patient with heart failure using telemonitoring and a computerised disease management system: a case report. *Eur J Cardiovasc Nurs* 2012;**11**:432–438.

118. Vries AE, Wal MHL van der, Nieuwenhuis MMW, Jong RM de, Dijk RB van, Jaarsma T, Hillege HL. Health professionals' expectations versus experiences of internet-based telemonitoring: survey among heart failure clinics. *J Med Internet Res* 2013;**15**.

119. Vuorinen A, et al. Use of home telemonitoring to support multidisciplinary care of heart failure patients in Finland: randomized controlled trial. *Journal Med Internet Res* 2014;**16**:1–12.

120. Wade M.J., Desai A.S., Spettell C.M., Snyder A.D., McGowan-Stackewicz V., Kummer P.J., Maccoy M.C., Krakauer R.S. Telemonitoring with case management for seniors with heart failure. *Am J Managed Care* 2011;**17**:e71–e79.

121. Wakefield B, Holman J, Ray A, Scherubel M, Burns T, Kienzle M, Rosenthal G. Outcomes of a home telehealth intervention for patients with heart failure. *J Telemed Telecare* 2009;**15**:46–50.

122. Weintraub A, Gregory D, Patel AR, Levine D, Venesy D, Perry K, Delano C, Konstam MA. A Multicenter Randomized Controlled Evaluation of Automated Home Monitoring and Telephonic Disease Management in Patients Recently Hospitalized for Congestive Heart Failure: The SPAN-CHF II Trial. *Journal of Cardiac Failure* 2010;**16**:285–292.

123. Whitten P, Bergman A, Meese MA, Bridwell K, Jule K. St. Vincent's Home telehealth for congestive heart failure patients. *Telemed J E Health* 2009;**15**:148–153.

124. Whitten P, Mickus M. Home telecare for COPD/CHF patients: outcomes and perceptions. *Journal of telemedicine and telecare* 2007;**13**:69–73.

125. Winkler S, Schieber M, Luecke S, Heinze P, Schweizer T, Wegertseder D, Scherf M, Nettlau H, Henke S, Braecklein M, Anker SD, Koehler F. A new telemonitoring system intended for chronic heart failure patients using mobile telephone technology - Feasibility study. *International Journal of Cardiology* 2011;**153**:55–58.

126. Woodend AK, Sherrard H, Fraser M, Stuewe L, Cheung T, Struthers C. Telehome monitoring in patients with cardiac disease who are at high risk of readmission. *Heart & Lung* 2008;**37**:36–45.

127. Wootton R, Gramotnev H, Hailey D. A randomized controlled trial of telephone-supported care coordination in patients with congestive heart failure. *J Telemed Telecare* 2009;**15**:182–186.

128. Wu RC, Delgado D, Costigan J, Maciver J, Ross H. Pilot study of an Internet patient-physician communication tool for heart failure disease management. *J Med Internet Res* 2005;**7**.
