# Supplementary figures and images for: Evaluation Criteria of Noninvasive Telemonitoring for Patients With Heart Failure: Systematic Review
Source: J Med Internet Res. 2018 Jan 16;20(1):e16. doi: 10.2196/jmir.7873 (PMC6257336; doi:10.2196/jmir.7873)

## Slide 1
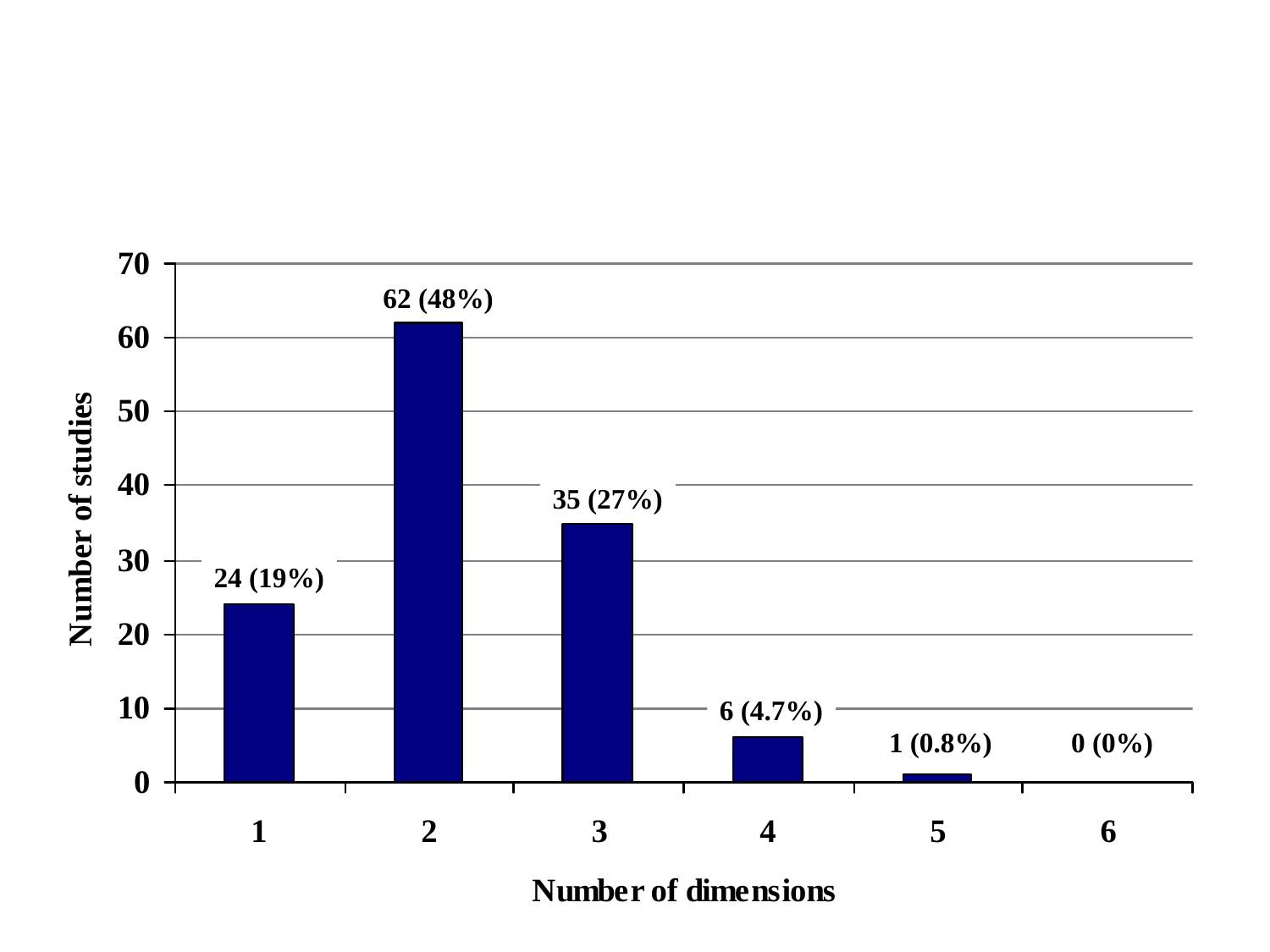

62 (48%)
35 (27%)
24 (19%)
6 (4.7%)
1 (0.8%)
0 (0%)

Supplement: Multimedia Appendix 5 [file jmir_v20i1e16_app5.ppt]

## Slide 1
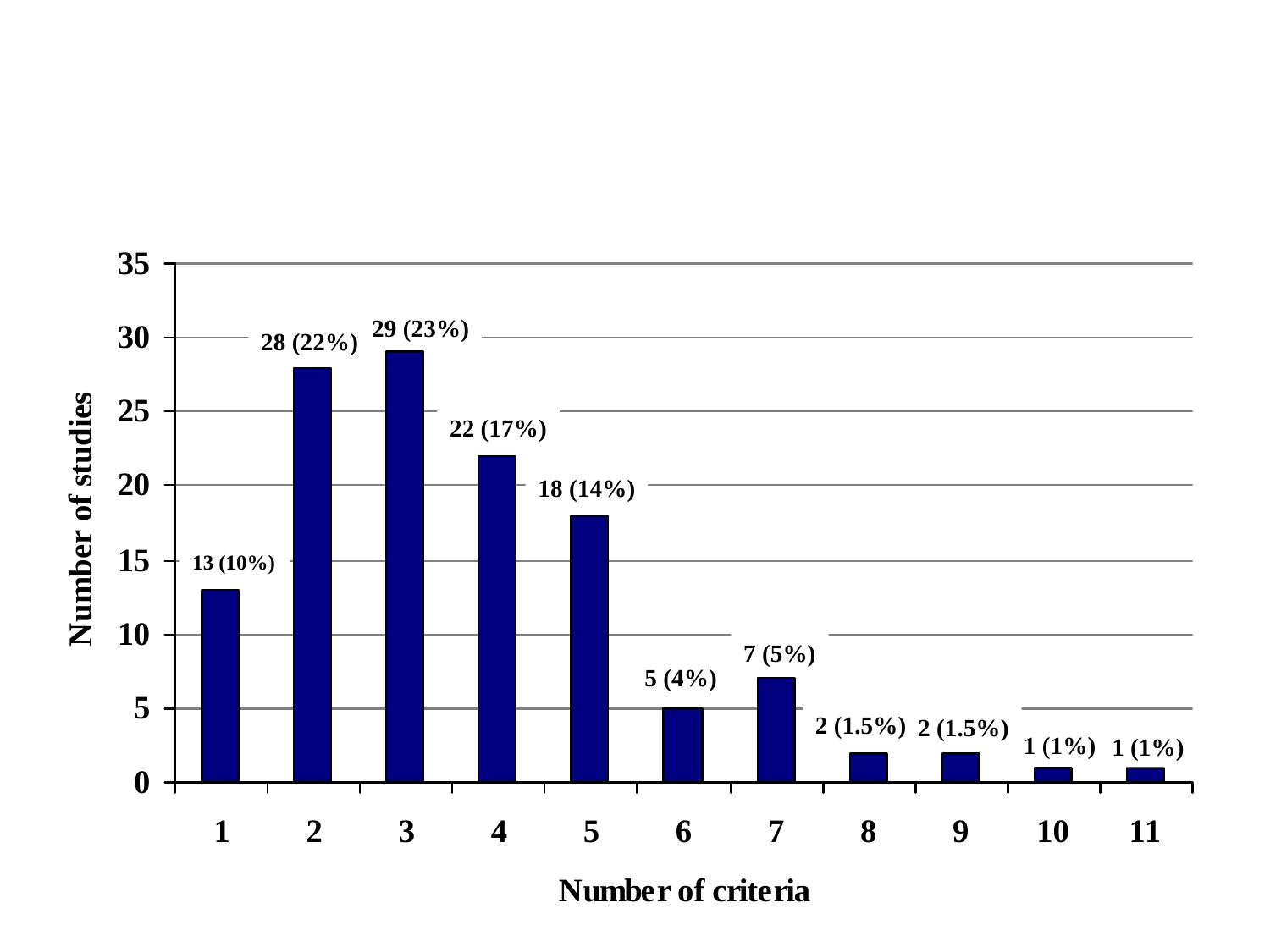

29 (23%)
28 (22%)
22 (17%)
18 (14%)
13 (10%)
7 (5%)
5 (4%)
2 (1.5%)
2 (1.5%)
1 (1%)
1 (1%)

Supplement: Multimedia Appendix 6 [file jmir_v20i1e16_app6.ppt]
